# The Association between Neoadjuvant Radio-Chemotherapy and Prolonged Healing of Anastomotic Leakage after Esophageal Resection Treated with EndoVAC Therapy

**DOI:** 10.3390/jcm11164773

**Published:** 2022-08-16

**Authors:** Philippa Seika, Matthias Biebl, Jonas Raakow, Nadja Berndt, Linda Feldbrügge, Max Magnus Maurer, Eva Dobrindt, Peter Thuss-Patience, Johann Pratschke, Christian Denecke

**Affiliations:** 1 Chirurgische Klinik, Campus Charité Mitte|Campus Virchow-Klinikum, Charité Universitätsmedizin Berlin, 13353 Berlin, Germany; 2 Department of Surgery, Division of Surgical Sciences, Beth Israel Deaconess Medical Center, Harvard Medical School, Boston, MA 02215, USA; 3 Medizinische Klinik mit Schwerpunkt Hämatologie, Onkologie und Tumorimmunologie, Campus Charité Mitte|Campus Virchow-Klinikum, Charité Universitätsmedizin Berlin, 13353 Berlin, Germany

**Keywords:** esophageal surgery, endoscopic vacuum therapy (EVT), anastomotic leakage

## Abstract

(1) Background: Endoscopic vacuum therapy (EVT) has become the mainstay in the treatment of early anastomotic leakage (AL) after esophageal resection. The effect of nRCT on the efficacy of EVT is currently unknown. (2) Methods: Data of 427 consecutive patients undergoing minimally invasive esophagectomy between 2013 and 2022 were analyzed. A total of 26 patients received EVT for AL after esophagectomy between 2010 and 2021. We compared a cohort of 13 patients after treatment with EVT for anastomotic leakage after neoadjuvant radiochemotherapy (nRCT) with a control group of 13 patients after neoadjuvant chemotherapy (nCT) using inverse propensity score weighting to adjust for baseline characteristics between the groups. EVT therapy was assessed regarding patient survival, treatment failure as defined by a change in treatment to stent/operation, duration of treatment, and secondary complications. Statistical analysis was performed using linear regression analysis. (3) Results: Time to EVT after initial tumor resection did not vary between the groups. The duration of EVT was longer in patients after nRCT (14.69 days vs. 20.85 days, *p* = 0.002) with significantly more interventions (4.38 vs. 6.85, *p* = 0.001). The success rate of EVT did not differ between the two groups (nCT *n* = 8 (61.54%) vs. nCT *n* = 5 (38.46%), *p* = 0.628). The rate of operative revision did not vary between the groups. Importantly, no mortality was reported within 30 days and 90 days in both groups. (4) Conclusions: EVT is a valuable tool for the management of AL after esophageal resection in patients after nRCT. While the success rates were comparable, EVT was associated with a significantly longer treatment duration. Anastomotic leakages after nRCT often require prolonged and multimodal treatment strategies while innovative strategies such as prophylactic endoVAC placement or use of a VAC-Stent may be considered.

## 1. Introduction

The incidence of esophageal cancer has been increasing in recent years [1] while for decades, surgical resection alone has been the standard treatment. However, even as surgical techniques have improved and postoperative mortality rates have declined, the overall survival (OS) has remained stagnant with surgery alone [2,3]. Nowadays, neoadjuvant RCT (nRCT) is an established modality in the treatment of esophageal cancer [4]. Radiation and chemotherapy lead to an impairment not only of cancer cells but also of the surrounding tissue [5,6]. Compromised wound healing in irradiated tissues is a common and challenging clinical problem in the management of surgical complications.

On the other hand, anastomotic leakage (AL) is one of the most severe surgical complications resulting in significant morbidity, prolonged hospital admission, and increased risk of mortality [7,8]. Treatment options include surgical revision (operative revision of anastomosis, cervical fistula), endoscopic stent therapy, and endoscopic vacuum therapy (EVT). In this regard, endoluminal negative-pressure therapy is highly effective, especially in patients with potentially damaged or hypoxic tissue, as it promotes changes in blood flow, decreases local edema, reduces bacterial contamination, and stimulates tissue healing due to mechanical stress [9,10,11]. Consequently, EVT has been established in the management of esophageal perforations and anastomotic leakage following upper gastrointestinal surgery [12,13]. With increasing expertise, the technique is associated with a range of anastomotic leakage closure of 66.7 to 100.0 percent [14]. The endoluminal sponge continuously drains fluid, thereby reducing the bacterial load of the perforation cavity and facilitating granulation. However, nowadays, combined with a nasogastric feeding tube, EVT is often associated with prolonged periods (>3 weeks) of parenteral nutrition, with patients reporting frustration and a reduced quality of life during the potentially long treatment period. In contrast, the application of a stent for AL treatment allows for the continuation of oral nutrition. As the EVT is a relatively new technique, there is currently no standardized guideline for the use or discontinuation of the treatment method. In this regard, the ESOLEAK Phase II clinical trial may provide important data in the use of endoscopic stent placement vs. EVT [15].

Whether neoadjuvant radiation poses a risk factor for the healing of anastomosis is currently debated. A recent study described no correlation between preoperative radiation and leakage rates of intrathoracic anastomoses [16]. In contrast, an interesting study by Juloori et al. reported a strong correlation of the field of irradiation and anastomotic healing [17], while a recent systematic review revealed conflicting data about radiation doses and the incidence of anastomotic leakage following esophagectomy [4]. Given a potential negative impact of the radiation field on any subsequent healing process, local EVT treatment may also be impaired. Therefore, we aimed to investigate the impact of a neoadjuvant treatment on the efficacy of EVT for anastomotic leakage after esophagectomy for esophageal cancer in a propensity-weighted analysis.

## 2. Materials and Methods

A prospectively maintained institutional database containing all patients undergoing resection for adenocarcinoma of the esophagus, gastroesophageal junction, or stomach was reviewed. Clinicopathological data of all 427 consecutive patients undergoing minimally invasive esophagectomy between 2013 and 2022 for esophageal cancer were evaluated. All patients with postoperative anastomotic leakage (AL) treated with primary endoluminal vacuum therapy were included. Resections were performed as either open, laparoscopic, hybrid, or robotic surgery procedures. Patients who underwent palliative surgery and patients with cancer of the GEJ or presenting with a second malignant disease at time of surgery or without neoadjuvant treatment were excluded. Furthermore, all patients with graft necrosis were excluded. A total of twenty-six patients were treated with EVT for anastomotic leakage after esophageal resection for a primary malignancy during the nine-year study period. We compared a cohort of thirteen patients after treatment with EVT for anastomotic leakage after nRCT with a control group of thirteen patients after nCT. The primary endpoint of this study was the success of EVT. Successful EVT was defined as a full defect closure while preserving gastrointestinal continuity. In the case of EVT discontinuation, the degree of response to EVT was further analyzed. Therefore, cases in which a reduction in size of the defect was achieved via EVT and followed by a synergistic secondary procedure and cases where no response was seen were also differentiated.

In the case of EVT discontinuation, the reason for the discontinuation of EVT was reported, e.g., change due to insufficient progress, progressive infection, or death. Additionally, the alternative treatment modality was reported including a change to stent therapy, operative revision of the anastomosis, or loss of esophageal continuity. Secondary endpoints were diagnostic parameters of AL in these groups, including changes in infection parameters (CRP, Leucocytes) during diagnosis, duration of treatment, the number of changes required and perioperative morbidity and mortality, as well as morbidity and mortality after a 30-month observation period.

### 2.1. Statistical Analysis

A comparison of preoperative patient’s characteristics by standardized differences who underwent minimally invasive esophagectomy (MIE) for esophageal cancer and AL following nCT or nRCT was performed. Subsequently, baseline imbalances were controlled with propensity scores and stabilized by inverse weighting. Propensity scores were estimated by logistic regression models with the type of neoadjuvant (nRCT vs. nCT) treatment as the dependent variable and pre-treatment characteristics, which exceeded 10% of the difference at univariate analyses as independent variables. These included the following matching parameters: sex, coronary heart disease, hypertension, and squamous cell carcinoma (SCC). After weighting, the standardized difference was computed for all variables to assess imbalance, and values less than 0.1 were considered balanced. Stabilized inverse weights equal to (1/propensity score of nRCT) and (1/1-propensity score of nCT) were assigned to nRCT and nCT patients, respectively. Finally, analysis was performed using a robust logistic regression model based on weighted data to assess the response to EVT treatment according to time to AL, additional complications, hospital duration, treatment duration, and treatment failure adjusting for baseline score. All analyses were two-sided, and *p* < 0.05 was considered significant. Analyses were performed using SPSS software, version 25 (SPSS Inc., Chicago, IL, USA).

### 2.2. Preoperative Assessment

As part of routine preoperative preparation, all patients underwent routine evaluation including medical history, physical examination, laboratory studies, imaging studies, and pre-anesthesia evaluation. Diagnosis and staging of esophageal cancer were obtained via esophagogastroduodenoscopy with multiple biopsies and endosonography. Staging was completed with cross-sectional imaging (computer tomography or magnetic resonance imaging) and diagnostic laparoscopy for adenocarcinomas. In some cases, fluorodeoxyglucose–positron emission tomography (PET) was performed to rule out metastatic disease.

### 2.3. Neoadjuvant Treatment

For each patient, treatment was recommended by a multidisciplinary tumor board. Preoperative treatment consisted of either chemotherapy (nCT) alone or combined radiochemotherapy (nRCT). In the nRCT cohort, patients received an induction therapy using GD 41.4 Gy, ED 1.8 Gy; followed by 5 weekly administrations of Carboplatin AUC2/Paclitaxel 50 mg/m^2^. In the nCT cohort, 12 patients received four cycles of pre-operative FLOT, while one patient received FLO.

### 2.4. Surgical Procedure

Transthoracic esophagectomy with gastric pull-up and two-field lymphadenectomy was performed on all patients. The procedures were conducted as previously reported [18]. Peritoneal metastases were ruled out by laparoscopy intraoperatively. In all cases, stapled circular end-to-side anastomoses were performed. To guide the stapler, an incision was created at the distal end of the gastric sleeve. A linear stapler was used to close the gastric incision. Finally, all patients received a chest tube while additional abdominal drains were implanted at the surgeon’s discretion. After surgery, all patients were admitted to a surgical intensive care unit for 2–3 days. Our current practice involves the removal of nasogastric tubes on the second postoperative day, as well as early mobilization and oral fluid intake, according to enhanced recovery after surgery protocols (ERAS). Prior to 2015, all patients had gastrointestinal decompression for five days following surgery, while the nasogastric tubes were removed after three days. In addition to anastomotic leakage, bleeding, intra-abdominal infections or wound infections, pneumonia, and organ failure were all reported as additional postoperative complications. Postoperative morbidity and mortality were defined as any complication (Clavien–Dindo 1–5) within 30 or 90 days after resection, respectively. The Clavien–Dindo classification of surgical complications was used to grade postoperative complications.

### 2.5. Histological Evaluation

The diagnosis of esophageal squamous cell carcinoma or adenocarcinoma was confirmed by histopathological examination prior to treatment. In addition, the tumor stage was determined using the TNM classification, and the surgical margins were intraoperatively evaluated for the absence of malignant cells (R0 resection).

### 2.6. Endoscopic Vacuum Therapy (EVT)

The application of EVT involved initial endoscopic evaluation to identify and characterize the wall defect and to evaluate the contaminated cavity. Once adequately evaluated, endoscopic irrigation and debridement, if applicable, was performed. Prior to sponge placement, a nasogastric tube was inserted into the stomach. The Endo-Sponge (B. BRAUN^®^, Melsungen, Germany) system was used in all cases. If necessary, a custom EVT sponge is assembled using a polyurethane foam (PUF). Depending on the size of the perforation, the sponge was either placed adjacent to the perforation site (if smaller than 10 mm) (intraluminal EVT) or placed through the perforation into the cavity (if larger than 10 mm) (intracavitary EVT). The vacuum therapy was applied at 125 mmHg of continuous pressure. Oral fluid intake in patients was restricted throughout the duration of EVT treatment. Sponges were changed every 3–4 days.

## 3. Results

Patient characteristics are shown in Table 1. There was a trend towards older patients in the nCT group (69.69 years vs. 62.38 years, *p* = 0.051). Patients in the nCT group also had a higher rate of arterial hypertension (nCT *n* = 10 (76.92%) vs. nRCT *n* = 4 (30.81%) *p* = 0.018) than patients receiving nRCT (Table 1). ASA scores were comparable between the groups with a trend towards higher scores in the nCT group (ASA 4, nCT *n* = 3 (23.08%) vs. nRCT *n* = 0 (0.00%), *p* = 0.148).

While both groups were comparable in terms of perioperative characteristics, comorbidity, and tumor histopathology, there was an increased number of patients presenting with squamous cell carcinoma in patients following nRCT (nCT *n* = 1 (7.7%) vs. nRCT *n* = 9 (69.23%) *p* = 0.001) (Table 2). Nodular involvement in preoperative staging (cN) was higher in the nRCT group (cN1; nCT *n* = 5 (38.46%) vs. nRCT *n* = 9 (69.23%) *p* = 0.015). In contrast, pTNM and UICC stage was comparable between the two groups (UICC stage 3; nCT *n* = 7 (53.85%) vs. nRCT *n* = 10 (76.92%) *p* = 0.413) (Table 2).

### 3.1. Diagnosis of Anastomotic Leakage

The defect size (cm) at initial diagnosis did not differ significantly between the two groups (nCT, 13.69 mm vs. nRCT 19.58 mm, *p* = 0.467) (Table 3). There was no significant difference in changes in C-Reactive protein one day prior to AL diagnosis between the two groups (C-Reactive protein (CRP, mg/L) (nCT 182.34 mg/L vs. nRCT 153.70 mg/L, *p* = 0.232). Contrastingly, leucocyte values were significantly lower in the nRCT group on the day prior to AL diagnosis and on the day of diagnosis (leucocytes (Table 3).

### 3.2. EVT Treatment

**Figure 1:**
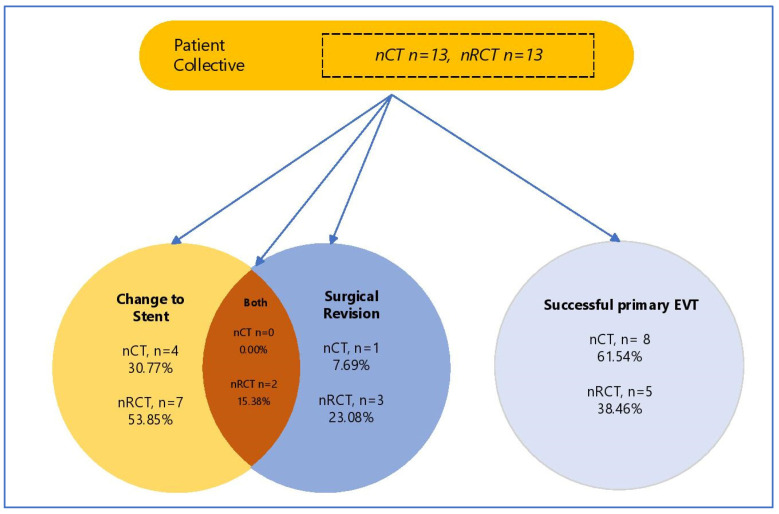
Study population according to treatment modality of AL. EVT—EndoVAC Therapy, nCT—neoadjuvant chemoptherapy, nRCT—neoadjuvant chemoradiotherapy.

A complete closure of the defect was achieved by EVT alone in 61.54% of patients after nRCT and 38.46% of patients after nCT (successful primary EVT; nCT *n* = 5 (38.46%) vs. nRCT *n* = 8 (61.54% (*p* = 0.628) (Table 3, Figure 1). A synergistic approach, whereby a decrease in size was achieved via EVT with subsequent stenting, was successful in 84.61% of patients in both groups (*n* = 11 (84.61%) vs. *n* = 11 (84.61%), *p* = 0.999). Finally, two patients in each group showed no discernible decrease in size after EVT (nCT: *n* = 2 (15.38%) vs. nRCT: *n* = 2 (15.38%), *p* = 0.696). There was a significantly longer duration of EVT treatment in the nRCT group (nCT 14.69 days vs. nRCT 20.85 days, *p* = 0.002) with a significantly higher number of endoscopic interventions required (nCT 4.38 vs. nRCT 6.85, *p* = 0.001). This, however, did not coincide with a longer duration of hospital admission (nCT 77.85 days vs. nRCT 53.69 days, *p* = 0.304). Patients after nRCT showed a trend towards a more frequent change in the treatment modality to stent therapy (nCT *n* = 4 (30.77%) vs. nRCT *n* = 7 (53.85%) *p* = 0.518); this was, however, not statistically significant (Figure 1). Operative revision of the anastomosis after EVT occurred at the same rate in the nRCT both groups (nCT *n* = 1 (7.69%) vs. nRCT *n* = 3 (23.08%) *p* = 0.764). There was no incidence of loss of oesophageal continuity in our cohort.

### 3.3. Perioperative Complications

All patients in our collective suffered from an anastomotic leakage (Table 3). Concomittent anastomotic stenosis occurred in one (7.69%) patient after nCT and two patients after nRCT (15.38%) (*p* = 0.401). Delayed gastric emptying occurred in five patients after nCT (38.46%) and five patients after nRCT (30.77%) (*p* = 0.640). The incidence of SSI did not vary between the groups (nCT *n* = 2(15.38%) vs. nRCT *n* = 3 (23.08%), *p* = 0.775). There was an increased rate of perioperative pneumonia in the nRCT group (nCT *n* = 6 (46.15%) vs. nRCT *n* = 9 (61.23%), *p* = 0.030).

### 3.4. EVT Failure

As shown in Table 3 and Table 4, the defect becomes comparably smaller in both groups, but this took significantly longer after radiation. Therefore, the procedure was eventually discontinued (nCT *n* = 5 (38.46%) vs. nRCT *n* = 8 (61.54%), *p* = 0.628) despite a reduction in size. The reduction in size was comparable in both groups, suggesting that EVT was not ended prematurely in either group. There was no difference between the groups in terms of the estimated size of the defect when the decision was made to end EVT (nCT 11.75mm vs. nRCT 14.86mm, *p* = 0.762). Discontinuation of EVT was attributed to an insufficient therapeutic benefit (no decrease in size of the defect) in two patients in the nCT group and two patients in the nRCT group (nCT *n* = 2 (15.38%) vs. nRCT *n* = 2 (15.38%), *p* = 0.696). The presence or progression of perioperative complications was the reason for a change in therapeutic modality for AL. Two patients had generalized sepsis requiring intensive care management (*n* = 1 (7.69%) vs. *n* = 2 (15.38%), *p* = 0.696). In case of technical difficulties, endoscopic vacuum treatment was disrupted early on. This occurred in one patient in the nRCT group (*n* = 0 (0.00%) vs. *n* = 1 (7.69%), *p* = 0.696) (Table 4). No patients died during EVT therapy in either of the two groups.

### 3.5. Survival

**Figure 2:**
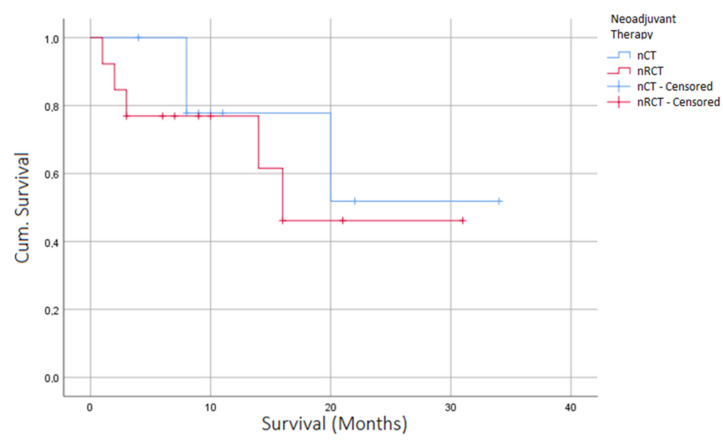
Kaplan–Meier curve depicting overall survival of patients after EVT for AL, stratified according to neoadjuvant therapy (nRCT/nCT).

The perioperative 30 day mortality (nCT *n* = 0 (0.00%) vs. nRCT *n* = 1 (7.14%), *p* = 0.131) and 90 day mortality (nCT *n* = 0 (0.00%) vs. nRCT *n* = 1 (7.14%), *p* = 0.131) did not differ between the groups despite a significant difference in terms of tumor entity between the groups (SCC (*n* = 1, 7.69%) vs. nRCT (*n* = 9, 69.23%), *p* = < 0.001). With a follow up time of 30 months, no differences in overall survival were seen between the two groups (Figure 2). The median overall survival was 19.4 months for patients with nCT compared to 16.8 months for patients with nRCT-treated tumors (*p* = 0.864, Figure 2).

## 4. Discussion

While there is a benefit in using neoadjuvant radiochemotherapy in terms of survival, as demonstrated by the CROSS-Trial [3], radiation may pose a challenge in terms of the management of perioperative complications [6]. To prevent the development of leakage, early removal of the nasogastric tube and omentoplasty have been suggested recently as significant measures [18]. However, once a leak is detected, urgent treatment is required to prevent septic complications.

While data regarding the effect of preoperative radiochemotherapy on the incidence of anastomotic leakage are not conclusive, the literature suggests that there is a relationship between radiation and the incidence of AL. A study by Juloori et al. correlated the preoperative radiation field (50.4 Gy) with the postoperative location of the anastomosis using computed tomography [17]. Anastomotic leakage rates were more than fourfold higher if the anastomosis was placed inside the former radiation field compared to anastomoses outside the former radiation field, thereby identifying radiation as a significant risk factor. It is unclear to what extent radiation dose may affect the incidence of AL. In a retrospective study of fifty-three patients, the authors found no significant relationship between the radiation dose and anastomotic leakage [19]. However, Bang et al. demonstrated that patients who experienced anastomotic complications after nRCT for esophageal cancer were more likely to have received a higher mean esophageal dose administered near the azygous vein [20]. While this may explain the discrepancy in the literature, it also highlights the complexity of decision-making involved in the treatment of esophageal cancer.

As such, the management of anastomotic leaks in this patient collective poses a unique challenge. Limited literature exists to guide treatment of AL in patients after radiation therapy. Endoscopic vacuum treatment for esophageal leaks and perforations has been reported in many publications, with most authors reporting high success rates and few procedural complications [21,22]. Most series feature a variety of pathologies and are not focused on anastomotic leaks [21]. Some authors report the superiority of EVT to other modalities [23,24], and EVT is therefore the primary treatment strategy for AL in our institution.

Despite a growing body of literature, no robust evidence-based treatment guidelines for the management of AL exist. Verstegen et. al. conducted a systematic review on the management of intrathoracic and cervical anastomotic leakage after esophagectomy for esophageal cancer. The authors included 19 retrospective cohort studies including 273 patients. They surmised that no evidence-based recommendations could be provided from the literature and therapy should be decided on a case-by-case basis [25]. In small AL without a cavity, endoscopic treatment with either stent or endoluminal vacuum therapy is possible [25]. While both endoscopic modalities show good clinical success, there are significant shortcomings inherent in each technique, leading to increasing applications of combined approaches. Stenting is a viable option in small defects and maintains patency of the esophageal lumen facilitating enteral feeding. However, a larger defect size, ischemic esophageal tissue, or sepsis are factors that drastically decrease the clinical success of stent therapy [21,26]. Due to the performance of EVT in ischemic or damaged tissue, it has been used as a preventive therapy in ischemic gastric tube after esophagectomy [27]. Further combined applications include the recent implementation of VacStent therapy, which maintains the patency of the esophageal lumen whilst providing the tissue regenerative properties associated with EVT [28]. While AL is increasingly treated endoscopically, surgical revision should be considered in certain cases. Indications for surgical re-intervention include the severity of symptoms and condition of the patient, failure of initial treatment (step up approach), or extended anastomotic disruption. Surgical options include suturing of the anastomosis, cervical fistula, colonic interposition (primary or as a two-stage procedure), or jejunum interposition [29].

In the present study, we found that EVT is suitable in the treatment of AL in patients after both nCT and nRCT. The rates of successful EVT across both groups is comparable to the literature, albeit in the higher range. Berlth et al. analyzed the outcomes of 111 patients with esophageal defects managed by stent placement or endoscopic vacuum therapy; unlike in our present study, various etiologies were included. Successful closure of defects was achieved in 85.7% of patients with endoscopic vacuum therapy [30]. Again, this success rate of stent therapy does not bear comparison with previously published rates. Virgilio et al. conducted a review of 29 studies (17 retrospective, 6 prospective, and 6 case reports) with a total of 209 patients. The authors found the range of anastomotic leakage closure to be between 66.7 and 100% [14]. As a tertiary referral center, our nCT cohort may have consisted of patients with more comorbidity affecting wound healing such as a high percentage of cardiac diseases and patients classified as ASA 3 and 4. This was disproportionately the case in the cohort after nCT, suggesting that the failure rates in this cohort may be higher than expected. However, cardiovascular comorbidity was controlled for in weighting. The comparatively higher failure rate in the nRCT group occurred despite similar initial defect sizes, and comparable defect sizes when treatment modality was changed. A further explanation for this discrepancy in terms of the success rate is the variation of definitions of a successful defect closure. Some authors only consider a success in EVT as a full closure of the defect while others also classify a synergistic therapeutic approach with EVT in combination with other modalities such as either an OTSC clip or stent as a success of EVT. In the present study, we report our results according to both common definitions.

In the present study, we showed a correlation between irradiation and prolonged healing of AL by EVT despite comparable closure rates. EVT was discontinued more frequently in patients after nRCT than nCT, although this was not statistically significant. In this case, it is a reasonable assumption that the increased incidence of EVT failure may be related to the increased perioperative morbidity associated with a longer duration of EVT. Change in treatment modality was often due to new-onset infection such as pneumonia rather than an insufficient defect closure. There was a significantly higher incidence of pneumonia in the nRCT group. When reporting the reason for change in modality, more patients suffered from sepsis during EVT in the nRCT group compared to the nCT group. The deterioration of the patient condition under therapy and a resulting change in modality was reported more commonly. Furthermore, endoscopic vacuum therapy was also disrupted in one patient after nRCT at an early stage due to technical problems (insufficient sealing). This was not seen in the nCT group. The decision was made to discontinue EVT to avoid extended periods of intensive care, hospitalization, and increased rates of nosocomial infection associated with delayed defect closure. Therefore, despite a comparable reduction in size of the defect, EVT was discontinued in these patients more often and another procedure was chosen. A study by Shubert et al. correlated the size of the defect with the success of stent therapy, suggesting that larger perforations can be treated with stent placement if the dehiscence of the lumen circumference does not exceed 70% [31]. As such, EVT was the first step in a multimodal concept to enable a stent in these cases.

To control for a possible pre-emptive discontinuation of EVT, we compared the defect sizes at the time of modality change. There was no difference in terms of a decrease in size of the defect between the two groups. Furthermore, EVT was a new modality being established throughout the course of our study; therefore, no guidelines govern the indication for the discontinuation of therapy. Therefore, while there is no difference in pre-emptive discontinuation of EVT between the two groups, it is possible that endoVAC treatment was discontinued too early in the earlier cases in the entire cohort as the optimal duration of treatment was not known. The learning curve of EVT was not controlled for in our study.

The pathophysiology of anastomotic healing after radiation, especially in the context of negative-pressure therapy, is still not fully understood. However, the current literature supports the relationship between irradiation and delayed wound healing. In irradiated tissue, there is a dysregulation of enzymes responsible for extracellular matrix synthesis (matrix metalloproteinase (MMP) and tissue inhibitors of metalloproteinases (TIMP)) resulting in disorganized collagen deposition by fibroblasts [6,32,33]. Experimental evidence shows that negative-pressure wound therapy may be particularly beneficial in facilitating the regeneration of damaged tissue. The physiological changes underlying the healing process in irradiated tissue indicate a decreased capacity for neovascularisation and increased fibrosis. Interestingly, numerous reports suggest a decrease in fibrosis and increased neovascularisation after cutaneous NPWT [34,35]. The molecular mechanism has, however, not been addressed in the context of nRCT or endoluminal negative-pressure therapy for AL.

Further limitations of this analysis include the small number of patients and the retrospective design of the study. Differences in patient population were seen, including increased cardiovascular comorbidity in the nCT group as well as increased SCC in the nRCT group. These differences were, however, controlled for in weighting. Despite these limitations, our findings might have clinical implications regarding combined treatments such as the combination of stent and EVT treatment.

## 5. Conclusions

In our analysis, we found a significantly longer duration of EVT in patients after nRCT compared to nCT. However, the success rate of EVT is unaffected by nRCT, which underscores the efficacy of the procedure in this patient population. No difference was seen in terms of hospital LOS, long-term preservation of esophageal continuity, and perioperative mortality between the two groups. EVT may therefore be an appropriate modality in patients after nRCT, despite the prolonged course of the healing process. Thus, anastomotic leakages after nRCT may require prolonged and multimodal treatment strategies.

## Figures and Tables

**Table 1:** Univariate analysis of patient characteristics and comorbidity.

			nCT (*n* = 13)			nRCT (*n* = 13)		
		*n*	(%)	Mean	*n*	(%)	Mean	*p*=
Sex	Male	11	84.62%		9	69.23%		0.352 ^a^
	Female	2	15.38%		4	30.77%		
Age at resection				69.69			62.38	0.051 ^b^
BMI in (kg/m^2^)				26.86			24.57	0.207 ^b^
Hb at resection (mg/dL)				12.59			11.19	0.060 ^b^
Operation type	Open	0	0.00%		2	15.38%		0.392 ^a^
	Laparoscopic	5	38.46%		5	38.46%		
	Hybrid	5	38.46%		5	38.46%		
	Robotic	3	23.08%		1	7.69%		
ASA score	2	6	46.15%		6	46.15%		0.148 ^a^
	3	4	30.77%		7	53.85%		
	4	3	23.08%		0	0.00%		
	0	3	23.08%		6	46.15%		
Cardiovascular disease		10	76.92%		7	53.85%		0.216 ^a^
Pulmonary disease		3	23.08%		3	23.08%		0.999 ^a^
Renal disease		3	23.08%		0	0.00%		0.066 ^a^
Diabetes		2	15.38%		3	23.08%		0.619 ^a^
Hyperlipidemia		1	7.69%		3	23.08%		0.277 ^a^
Arterial hypertension		10	76.92%		4	30.77%		0.018 ^a,^*
Coronary heart disease		5	38.46%		1	7.69%		0.063 ^a^
Heart failure		1	7.69%		0	0.00%		0.308 ^a^
Peripheral arterial occlusive disease		1	7.69%		0	0.00%		0.308 ^a^

^a^. Chi-Squared test by Pearson, *. The chi-square statistic is significant at the 0.05 level, ^b^. Two-sample *t*-test for difference of means, ASA—American Society of Anaesthesiologists, BMI—Body Mass Index, HB—Haemoglobin

**Table 2:** Univariate analysis of histopathological characteristics.

		nCT (*n* = 13)			nRCT (*n* = 13)		
		*n*	(%)	*n*		(%)	*p*=
Tumor size in preoperative staging (cT)	cT 1	2	15.38%	0		0.00%	0.274 ^a^
	cT 2	3	23.08%	1		7.69%	
	cT 3	7	53.85%	11		84.62%	
	cT 4	1	7.69%	1		7.69%	
Tumor size in Histopathological evaluation (pT)	pT 0	4	30.77%	5		38.46%	0.416 ^a^
	pT 1	5	38.46%	2		15.38%	
	pT 2	1	7.69%	2		15.38%	
	pT 3	3	23.08%	2		15.38%	
	pT 4	0	0.00%	2		15.38%	
Nodular Involvement in preoperative staging (cN)	cN 0	4	30.77%	1		7.69%	0.015 ^a^
	cN 1	5	38.46%	9		69.23%	
	cN 2	2	15.38%	1		7.69%	
	cN 3	2	15.38%	2		15.38%	
Nodular involvement in histopathological evaluation (pN)	pN 0	9	69.23%	9		69.23%	0.999 ^a^
	pN 1	2	15.38%	2		15.38%	
	pN 3	2	15.38%	2		15.38%	
Differentiation (G)	G 0	1	7.69%	0		0.00%	0.687 ^a^
	G 1	1	7.69%	0		0.00%	
	G 2	8	61.54%	5		38.46%	
	G 3	2	15.38%	2		15.38%	
Resection margins (R)	R 0	12	92.31%	13		100.00%	0.347 ^a^
	R 1	1	7.69%	0		0.00%	
Lymphatic Invasion (L)	L 0	12	92.31%	10		76.92%	0.619 ^a^
	L 1	1	7.69%	3		23.08%	
Vascular Invasion (V)	V 0	12	92.31%	13		100.00%	0.308 ^a^
	V 1	1	7.69%	0		0.00%	
UICC Score	1	2	15.38%	0		0.00%	0.413 ^a^
	2	2	15.38%	2		15.38%	
	3	7	53.85%	10		76.92%	
	4	2	15.38%	1		7.69%	
G1-G3 Adenocarcinoma (%)		12	92.31%	4		30.77%	0.001 ^a,^*
SCC (%)		1	7.69%	9		69.23%	0.001 ^a,^*

^a^. Chi-Squared test by Pearson, *. The chi-square statistic is significant at the 0.05 level., cT—tumor size in preoperative staging, cN—nodular involvement in preoperative staging, G—Differentiation, L—Lymphatic Invasion, pT—Tumor size in Histopathological evaluation, pN—Nodular involvement in histopathological evaluation, R—Resection margins, SCC—Squamous cell carcinoma, UICC Score—International Union Against Cancer Score, V—Vascular Invasion

**Table 3:** EVT therapy and perioperative complications after esophageal resection.

			nCT (*n* = 13)			nRCT (*n* = 13)		
		*n*	(%)	Mean	*n*	(%)	Mean	*p*=
Diagnosis of AL	Days to diagnosis (POD)			8.62			10.93	0.294 ^b^
	Change in drain secretion	9	69.23%		9	69.23%		0.680 ^a^
	Initial defect size (mm)			13.69			19.58	0.467 ^b^
Infect parameters 1-day prior to AL	CRP (mg/L)			182.34			153.70	0.232 ^b^
	Leucocytes (×10^3^/µL)			11.93			9.68	<0.001 ^b,^**
Infect parameters on day of AL	CRP (mg/L)			204.56			179.41	0.200 ^b^
	Leucocytes (×10^3^/µL)			13.83			10.42	<0.001 ^b,^**
	Successful primary EVT	8	61.54%		5	38.46%		0.628 ^a^
	Number of sponges needed			4.38			6.85	0.001 ^b,^**
	Length of EVT treatment (days)			14.69			20.85	0.002 ^b,^**
	Duration of admission (LOS, days)			77.85			53.69	0.304 ^b^
	EVT failure (change to stent) ***	4	30.77%		7	53.85%		0.518 ^a^
	EVT failure (surgical revision of the anastomosis) ***	1	7.69%		3	23.08%		0.764 ^a,^*
	EVT failure (surgical revision: loss of esophageal continuity) ***	0	0.00%		0	0.00%		
	EVT failure (more than one change in treatment)	0	0.00%		2	15.38%		0.253 ^a^
Perioperative Morbidity	30-day mortality	0.00	0.00%		0.00	0.00%		
	90-day mortality	0.00	0.00%		0.00	0.00%		
Perioperative Complications	Anastomotic stenosis	1	7.69%		2	15.38%		0.401 ^a^
	Delayed gastric emptying (DGE)	5	38.46%		5	30.77%		0.640 ^a^
	SSI	2	15.38%		3	23.08%		0.775 ^a^
	Pneumonia	6	46.15%		9	61.23%		0.030 ^a,^*
	Sepsis	5	38.46%		4	23.08%		0.107 ^a^
	Cardiovascular Complications	5	38.46%		4	23.08%		0.084 ^a^
	Neurological Complications	5	38.46%		4	23.08%		0.188 ^a^

^a^. Chi-Squared test by Pearson, *. The chi-square statistic is significant at the 0.05 level. ^b^. Two-sample *t*-test for difference of means, **. The *t*-test statistic is significant at the 0.05 level. *** multiple changes in modality possible. AL—anastomotic leakage, CRP—C-reactive protein, DGE—delayed gastric emptying, EVT—EndoVAC Therapy, LOS—length of stay, nCT—neoadjuvant chemoptherapy, nRCT—neoadjuvant chemoradiotherapy, POD—postoperative day, SSI—surgical site infection

**Table 4:** EVT failure and reasons for change in modality.

			nCT (*n* = 13)			nRCT (*n* = 13)		
		*n*	(%)	Mean	*n*	(%)	Mean	*p*=
Successful primary EVT		8	61.54%		5	38.46%		0.628 ^a^
Reduction in size of the defect via EVT		11	84.61%		11	84.61%		0.999 ^a^
Estimated size of defect (initial, mm)				13.69			19.58	0.383 ^b^
Estimated size of defect at change of modality (mm)				11.75			14.86	0.762 ^b^
Estimated change in size at change in modality (mm)				23.50			17.00	0.550 ^b^
Estimated change in size at change in modality (%)				54.36%			40.26%	0.555 ^b^
Reason for end of primary EVT	Insufficient decrease in size	2	15.38%		2	15.38%		0.696 ^a^
	Sepsis	1	7.69%		2	15.38%		
	Patient deterioration	0	0.00%		1	7.69%		
	Synergistic therapy	2	15.38%		2	15.38%		
	Technical problems	0	0.00%		1	7.69%		

^a^. Chi-Squared test by Pearson, ^b^. Two-sample *t*-test for difference of means, EVT—EndoVAC Therapy.

## Data Availability

Data available from authors upon request.
